# Host-specific phenotypic variation of a parasite co-introduced with invasive Burmese pythons

**DOI:** 10.1371/journal.pone.0209252

**Published:** 2019-01-02

**Authors:** Aundrea K. Westfall, Melissa A. Miller, Christopher M. Murray, Bryan G. Falk, Craig Guyer, Christina M. Romagosa

**Affiliations:** 1 Department of Biological Sciences, Auburn University, Auburn, Alabama, United States of America; 2 US Geological Survey, Fort Collins, Colorado, United States of America; 3 Wildlife Ecology and Conservation, University of Florida, Gainesville, Florida, United States of America; University of Pretoria, SOUTH AFRICA

## Abstract

Invasive Burmese pythons (*Python bivittatus* Kuhl, 1820) have introduced a lung parasite, *Raillietiella orientalis*, (Hett, 1915) from the python’s native range in Southeast Asia to its introduced range in Florida, where parasite spillover from pythons to two families and eight genera of native snakes has occurred. Because these novel host species present a diversity of ecological and morphological traits, and because these parasites attach to their hosts with hooks located on their cephalothorax, we predicted that *R*. *orientalis* would exhibit substantial, host-associated phenotypic plasticity in cephalothorax shape. Indeed, geometric morphometric analyses of 39 parasites from five host species revealed significant variation among host taxa in *R*. *orientalis* cephalothorax shape. We observed differences associated with host ecology, where parasites from semi-aquatic and aquatic snakes exhibited the greatest morphological similarity. Morphological analyses of *R*. *orientalis* recovered from invasive pythons, native pit vipers, and terrestrial snakes each revealed distinct shapes. Our results suggest *R*. *orientalis* can exhibit significant differences in morphology based upon host species infected, and this plasticity may facilitate infection with this non-native parasite in a wide array of novel squamate host species.

## Introduction

Non-native species can harbor parasites and pathogens capable of infecting native taxa within their introduced range, a process known as parasite spillover [1–3]. For species with indirect lifestyles, potential obstacles to parasite spillover include low host density and lack of an appropriate intermediate host [4]. When a parasite species with an indirect life cycle successfully establishes, it may remain host-specific, infecting only the non-native host with which it was introduced, so long as an appropriate intermediate host is present [5]. This may result from low host susceptibility among potential definitive hosts within the parasite’s invaded range, a lack of appropriate intermediate hosts to allow transfer to potential hosts, or from founder effects resulting in reduced plasticity preventing transfer pathways present in the indigenous range of the parasite [6]. Despite the complexity of indirect parasite life cycles, some parasites co-introduced with non-native hosts have demonstrated an ability to infect novel hosts native to the introduced range [7, 8], often due to a combination of a parasite’s phenotypic plasticity among hosts [9–12] and the immunological naivety of a new host, rendering the host unable to deter infection by the novel parasite [13, 14].

Burmese pythons (*Python bivittatus*), native to Southeast Asia, have become established in southern Florida [15, 16] where they have co-introduced a lung parasite (*Raillietiella orientalis*: Raillietiellidae) previously unknown from North America [3]. *Raillietiella orientalis* has spilled over into the assemblage of native Floridian snakes, where the parasites have higher prevalence and intensity, achieve larger body size, and have populations dominated by reproductive females (Miller *et al*., in review). Within its native Asian distribution, *R*. *orientalis* infects snakes of at least four families, likely because a variety of intermediate hosts can be used to complete the indirect life cycle of this parasite [17]. Thus, the diverse network of intermediate and definitive hosts of the parasite in its native range appears to be replicated in Florida [3].

The diversity of definitive hosts for *R*. *orientalis*, and the associated differences in the lungs of these hosts that vary significantly in form and function [18], may result in concomitant variation of the cephalothorax morphology of this parasite, since the cephalothorax contains the hook structures that allow attachment within the host’s lung. Paradoxically, all *R*. *orientalis* sampled from Florida thus far are a single species with limited genetic diversity [3], suggesting that any morphological adaptations that facilitate attachment to such a wide range of hosts will take the form of morphological (*i*.*e*., phenotypic) plasticity. We used geometric morphometric analyses, which have been used to demonstrate phenotypic variability among populations of other parasite species [19–22], to investigate plastic responses of *R*. *orientalis* to phylogenetically and ecologically divergent native host snakes in southern Florida. We used these data to aid in understanding the mechanism that allows this parasite to successfully infect a wide range of native host taxa (Miller *et al*., in review).

## Materials and methods

Burmese pythons were collected from their introduced range in southern Florida (Miami-Dade and Monroe Counties) during 2009–2015. Pythons were collected by hand during road surveys, opportunistic encounters, and through a collaborative removal effort between the United States Geological Survey and the National Park Service. Pythons were euthanized in accordance with AVMA guidelines by captive bolt gun and frozen within 24 hours. Native snakes were salvaged as road-kill during road surveys conducted in locations sympatric with pythons (see methods in [3]). Nocturnal road surveys were conducted consecutively, and salvaged native snakes were judged to be less than 24 hr post mortem upon collection and were immediately frozen. Snakes were dissected and pentastomes were collected and stored in 95% ethanol per standard preservation methods [23]. Methods of collection and preservation of snakes and parasites were comparable, respectively; Therefore, we assume potential fixation effects occurred in an unbiased fashion within and among hosts allowing us to interpret variation among hosts as indicating biological differences and not fixation effects. Pentastomes were cleared using an 80% phenol solution for 12–24 hours prior to being photographed. Each pentastome was placed on its dorsum on a microscope slide with a cover slip affixed on top of the specimen. Only adult female pentastomes were used, with sex determined based upon the presence (male) or absence (female) of copulatory spicules. Photographs of the ventral hooks and oral cadre (*i*.*e*. chitinised structure on mouth) of each parasite were taken at 2x magnification using a Nikon Eclipse Ni-E microscope and a Nikon DS-Fi2 camera; a 1000 μm scale was added to each image using Nikon NIS-Elements AR imaging software.

**Fig 1:**
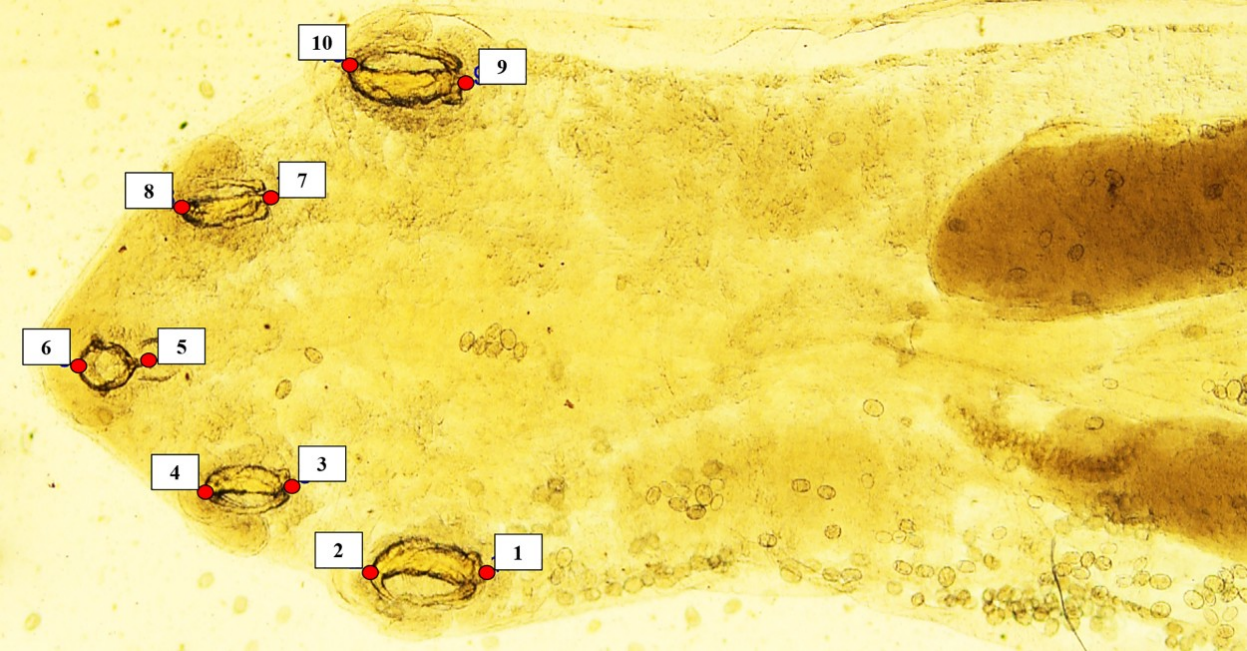
Whole mount of *Raillietiella orientalis* showing placement of homologous landmarks. Ten landmarks were used in geometric morphometric analyses to examine variation in hook and oral cadre morphology among host taxa. Pentastome samples were cleared in a phenol solution prior to analyses. The *R*. *orientalis* pentastome shown was collected from a snake, *Nerodia clarkii*, native to Florida.

Ten homologous landmarks were designated on each photograph to examine placement of the oral cadre and hooks used to grasp the host’s lung tissue during feeding (Fig 1); points 1, 3, 7, and 9 were at the insertion of each hook; points 2, 4, 8, and 10 were at the anterior-most point of the curve of each hook; point 5 was at the peg-like extension of the oral cadre near the pharynx; and point 6 was at the anterior-most point of the oral cadre. Landmarks from each photo were digitized and a tps file containing X, Y coordinates for each landmark was prepared using tpsDig2 software [24]. We used host species as a classifier variable and a Procrustes ANOVA was used to test for differences in parasite cephalothorax shape among host taxa. Canonical variates analyses (CVA) was used to visualize separation among parasites in multivariate space. Confidence ellipses (95%) were assigned to all ordinations. Centroid size variation among parasites was analyzed to assess the effect of variation in pentastome length on hook arrangement. Morphological variation of hook structure in *R*. *orientalis* was compared to a consensus specimen and was visualized in Cartesian space via transformation grids. All analyses were performed using MORPHOJ software [25].

## Results

A subsample of snake hosts collected were used in analyses to ensure the number of *R*. *orientalis* obtained from individuals of each host species met requirements for geometric morphometric analysis in which the total sample size minus the number of groups is greater than the number of landmarks [26]. Hosts of *R*. *orientalis* included five snake species, four of which were native to Florida (*Agkistrodon piscivorus* Gloyd, 1969: Viperidae; *Coluber constrictor* Linnaeus, 1758: Colubridae; *Nerodia clarkii* Kennicott, 1860: Colubridae; and *Thamnophis sirtalis* Linnaeus, 1758: Colubridae) and one snake that is a Florida invasive (*Python bivittatus*: Pythonidae). Thirty-nine *R*. *orientalis* specimens were examined from the five host species (Table 1).

**Table 1:** Sample sizes of snake hosts and pentastomes (*Raillietiella orientalis*) collected from southern Florida. The number of host individuals and the total number of parasites examined per host are shown for each host species. Snake species native to Florida included *Agkistrodon piscivorus*, *Coluber constrictor*, *Nerodia clarkii*, and *Thamnophis sirtalis*; *R*. *orientalis* collected from invasive Burmese pythons (*Python bivittatus*) were examined for comparison.

Host species	Number of host individuals	Number of *R*. *orientalis*
*Python bivittatus*	5	10
*Agkistrodon piscivorus*	2	4
*Coluber constrictor*	2	6
*Nerodia clarkii*	3	14
*Thamnophis sirtalis*	3	5
Total	15	39

**Fig 2:**
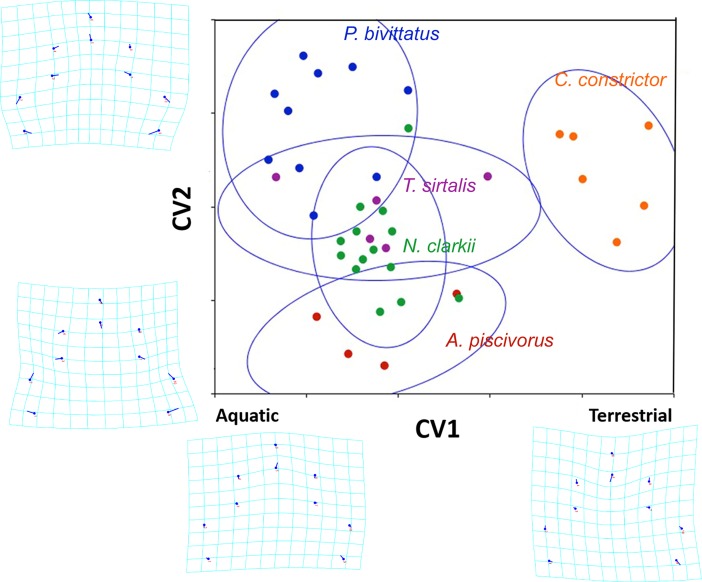
Canonical variance analysis plot depicting the relative separation of *Raillietiella orientalis* among snake hosts. Circles represent *R*. *orientalis* specimens obtained from their respective snake host (*Python bivittatus* = blue; *Thamnophis sirtalis* = purple; *Nerodia clarkii* = green; *Coluber constrictor* = orange; *Agkistrodon piscivorus* = red). Axis [CV1(x)] was replaced by a perceived biological axis based on ecological variation among hosts. Confidence ellipses (95%) are shown for each centroid. Wire frame grids are shown at the ends of each axis to provide context on variation in head morphology of *R*. *orientalis* along axes.

Parasite hooks and oral cadre morphology showed significant shape variation (Procrustes F = 2.27; df = 64; P = 0.0007). Centroid size variation was not significant (F = 2.16; df = 4; P = 0.09). Canonical variance analysis (CVA) separated terrestrial (*C*. *constrictor*) from aquatic (all other species) snakes along axis CV1(x) and separated pythonids (*P*. *bivittatus*) from pit vipers along axis CV2(y) (Fig 2). The total variation explained by both axes was 80.50%, with CV1(x) and CV2(y) accounting for 53.72% and 26.78% of the total variation, respectively.

**Fig 3:**
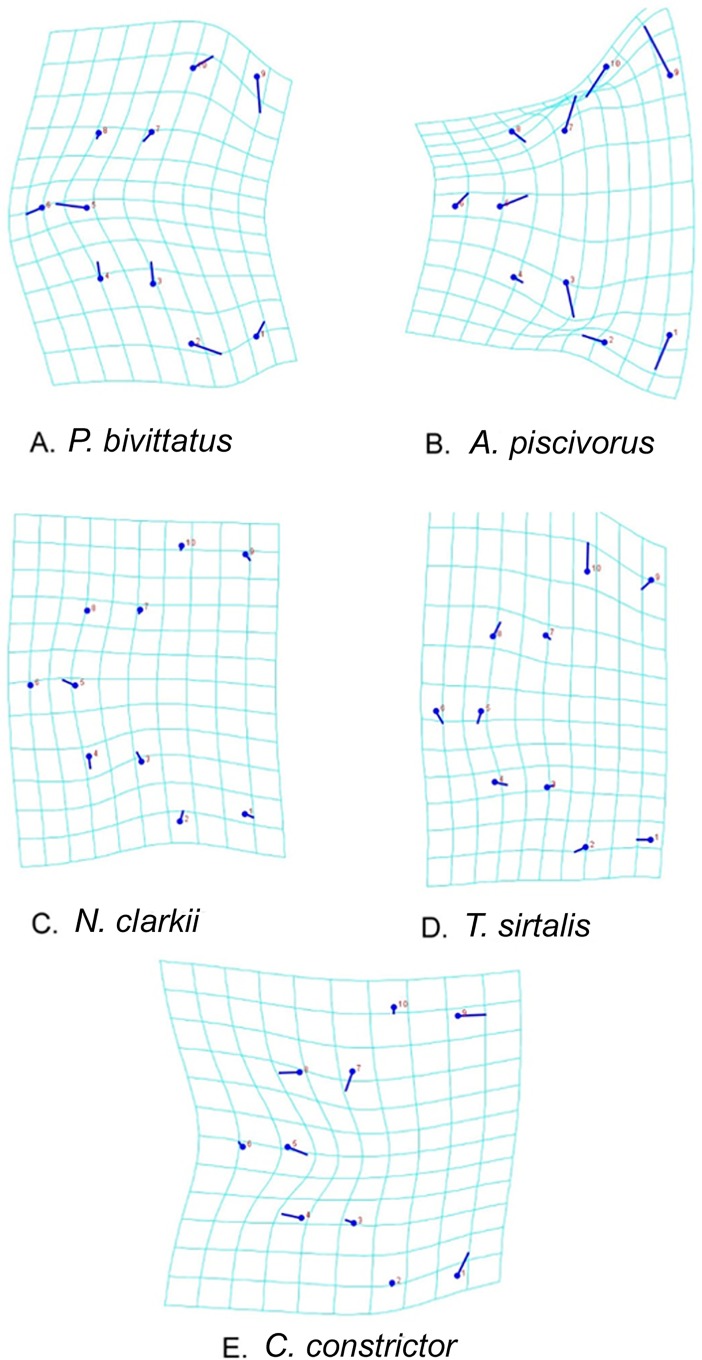
Transformation grids showing host-specific changes in hook and oral cadre morphology of *Raillietiella orientalis*. Morphological variation of *R*. *orientalis* among host species is shown by lines originating from a circle. Circles indicate the position of the average specimen. The direction of the line relative to a circle indicates specific variation in morphology among species relative to a consensus specimen.

Significant variation in pentastome cephalothorax morphology occurred in length of the oral cadre and the extension of the oral cadre near the pharynx, hook length, and rotation of the hooks (Fig 3). *Raillietiella orientalis* samples collected from pythons had the shortest oral cadre, parallel anterior hooks, and posterior hooks that rotated away from the midline of the body compared to *R*. *orientalis* collected from other snake hosts. Those from *A*. *piscivorus* exhibited wider placement of the posterior hooks as well as compression along the anteroposterior axis, causing the oral cadre to align with the anterior hooks laterally. The anterior point of each hook also turned medially, a feature unique to *R*. *orientalis* within this host. *Raillietiella orientalis* recovered from *N*. *clarkii* and *T*. *sirtalis* did not differ from each other in morphology, exhibiting comparable anteroposterior compression to *A*. *piscivorus*, but with hooks that were parallel along the body axis or turned slightly in a lateral direction. However, increased lateral rotation of the hooks was observed in *T*. *sirtalis* compared to *N*. *clarkii*. *Raillietiella orientalis* infecting *C*. *constrictor* exhibited the most compression along the long axis of the body, the longest oral cadre parallel anterior hooks and laterally-rotated posterior hooks.

## Discussion

All pentastomes used in this study were identified as *R*. *orientalis* based on phylogenetic analyses of the COI and 18S genes [3]. However, geometric morphometric analyses revealed morphologically distinct parasites according to their host taxa. The greatest similarities observed were exhibited by *R*. *orientalis* collected from *N*. *clarkii* and *T*. *sirtalis*, hosts that are morphologically, ecologically, and phylogenetically similar [27]. *Raillietiella orientalis* recovered from *P*. *bivittatus*, *A*. *piscivorus*, and *C*. *constrictor* each occupied distinct regions of morphological space, with *C*. *constrictor* parasites displaying the greatest distinction from other host taxa in hook and oral cadre morphology.

The relationship of parasite morphology and host taxa observed on the CVA ordination plot is best explained by the functional group of the host taxa, with aquatic and semi-aquatic snakes (*A*. *piscivorus*, *N*. *clarkii*, *T*. *sirtalis*, and *P*. *bivittatus*) separated from a terrestrial snake host (*C*. *constrictor*) along axis CV1(x). Aquatic snakes may share similar lung morphology due to selective pressures (*e*.*g*. hydrostatic pressure and respiratory demands) experienced in an aquatic environment [28]. Ecological similarity amidst aquatic snakes may promote development of analogous oral cadre and hook arrangements in *R*. *orientalis* adults.

Significant morphological groupings such as those we observed are likely to have one of two explanations: (1) host immune response affects pentastome development, or (2) pentastomes exhibit morphological plasticity to take advantage of diverse hosts. Host-induced morphological variation has been documented by numerous studies, but little in the way of an explanation has materialized [9, 11, 12, 29–34]. A series of experimental infections performed on calves with the nematode *Ostertagia ostertagi* demonstrated hosts that had been previously infected, or were older, with a better-developed immune system, contained parasites with significantly underdeveloped vulval flaps [31–33]. Further study revealed that parasites were more likely to be underdeveloped if their predecessors had arrested development [31]. However, the strongest factor affecting parasite development was the hosts’ immune response.

Variation of *R*. *orientalis* morphology among host species is not likely a result of host immune response, as *R*. *orientalis* is highly competent (*i*.*e*. able to reproduce) in snake hosts from both aquatic and terrestrial functional groups. If a host immune response elicited a change in oral cadre and hook morphology, it would be assumed that altering morphology would mitigate infection and limit the success of the parasite within the host. To the contrary, *R*. *orientalis* exhibits higher prevalence, infection intensity, fecundity, and greater size in snakes native to Florida versus the pythons from which they were introduced which has facilitated the spread of this parasite to native Florida snakes northward of the current geographic range of invasive Burmese pythons (Miller *et al*., in review). Therefore, in addition to an ability of *R*. *orientalis* to exploit immunologically naïve hosts, this parasite can alter its morphology in ways that suggest optimization of its capacity to attach to novel taxa within its invaded range.

Other pentastome species have demonstrated significant variation according to their developmental stage. *Raillietiella indica* was considered a distinct species based entirely on morphology, but genetic work elucidated that it is an early instar of *Raillietiella frenatus* [9]. Because females included in our study were adults and centroid size variation was found to be insignificant (*i*.*e*. variation in the length of *R*. *orientalis* among hosts did not account for the observed taxon-specific parasite hook morphology), ontogenetic variation among pentastomes does not explain the morphological groupings of *R*. *orientalis* recovered.

While effort to minimize fixation effects were taken, the potential for fixation error remains a possibility [35, 36]. If a fixation error was present, these effects would be expected to be distributed in an unbiased fashion among parasites within and among hosts, allowing us to describe biological effects despite any fixation effects that may linger. Moreover, wire frame grids, added to our CVA analysis, support that significant morphological variation of *R*. *orientalis* among host taxa observed in this study are unlikely to be influenced by the potential for muscle contraction or distortion of morphological traits during fixation.

Parasites use cues from their microenvironment to alter traits and behaviors that maximize fitness [34]. Morphological modification in a parasite is a mechanism known to decrease host specificity and, as a result, increases rates of infection [29]. Morphologically distinct phenotypes have been documented for numerous parasite species and much of this phenotypic variation has been linked to host taxa [12, 29, 30, 37, 38]. For many taxa, morphologically distinct parasites assumed to represent several species have been resolved into a single species under molecular analyses, showing that parasites exhibit less co-evolution with a specific host than previously believed and instead optimize the ability to utilize a variety of hosts [10, 39], (but see [40]). *Raillietiella orientalis* demonstrates this phenomenon. Within their native distribution in Asia, known definitive hosts include snakes from diverse families, including Pythonidae, Colubridae, Elapidae, and Viperidae [17]. Within its introduced Florida range, *R*. *orientalis* has been documented to infect snakes from two families and eight genera (Miller *et al*., in review), along with two genera of non-indigenous lizards (MAM, pers. comm.). Within its introduced Australian range, in addition to native snakes, introduced cane toads (*Rhinella marina*) have been infected by this parasite [9, 41]. Remarkable phenotypic plasticity may allow *R*. *orientalis* to infect such a variety of hosts where it has been introduced. Because these hosts are not co-evolved with *R*. *orientalis*, their efficacy to resist or ameliorate infection may be reduced (naïve host syndrome, [13]), allowing this non-native pentastome to maximize resource use from its novel host. In addition to the capability of *R*. *orientalis* to infect diverse taxa, the species’ demonstrated ability to alter its morphology to successfully infect hosts increases its potential for negatively impacting a multitude of hosts.

## Supporting information

S1 File TPS dataset. A TPS datafile is provided including all *Raillietiella orientalis* examined in geometric morphometric analyses.
(PDF)

S2 File Auburn university museum of natural history catalog numbers are provided for pentastomes and their respective host. The catalog number is provided for *Raillietiella orientalis* pentastomes examined using geometric morphometric analyses. All *R*. *orientalis* specimens were deposited to the Auburn University Museum of Natural History (AUM). The host species of each parasite is provided. Pentastomes from the same host share the same catalog number with different individual parasites identified by letter.
(PDF)
