# Supplementary material for: Host-specific phenotypic variation of a parasite co-introduced with invasive Burmese pythons
Source: PLoS One. 2019 Jan 2;14(1):e0209252. doi: 10.1371/journal.pone.0209252 (PMC6314578; doi:10.1371/journal.pone.0209252)
Supplement: S1 File — A TPS datafile is provided including all Raillietiella orientalis examined in geometric morphometric analyses. (PDF) [file pone.0209252.s001.pdf]

Supporting Information_TPS file data

LM=10
509.00000 288.00000
406.00000 292.00000
350.00000 408.00000
277.00000 411.00000
256.00000 512.00000
198.00000 518.00000
365.00000 622.00000
291.00000 635.00000
556.00000 723.00000
452.00000 749.00000
IMAGE=28744_A_NerCla_female_2X_scale.tif
ID=28744_A_NerCla
SCALE=0.004878
LM=10
518.00000 320.00000
412.00000 340.00000
396.00000 414.00000
307.00000 416.00000
302.00000 524.00000
262.00000 525.00000
433.00000 600.00000
352.00000 607.00000
600.00000 633.00000
502.00000 664.00000
IMAGE=28744_B_NerCla_female_2X_scale.tif
ID=28744_B_NerCla
SCALE=0.004878
LM=10
428.00000 261.00000
320.00000 290.00000
293.00000 395.00000
213.00000 422.00000
231.00000 533.00000
177.00000 553.00000
357.00000 601.00000
282.00000 646.00000
538.00000 664.00000
435.00000 706.00000
IMAGE=28744_C_NerCla_female_2X_scale.tif
ID=28744_C_NerCla
SCALE=0.004878
LM=10
458.00000 283.00000
352.00000 316.00000
296.00000 425.00000
214.00000 451.00000
206.00000 585.00000

145.00000 598.00000
355.00000 673.00000
272.00000 684.00000
562.00000 709.00000
455.00000 733.00000
IMAGE=28744_D_NerCla_female_2X_scale.tif
ID=28744_D_NerCla
SCALE=0.004878
LM=10
459.00000 217.00000
350.00000 202.00000
315.00000 295.00000
231.00000 299.00000
225.00000 406.00000
144.00000 395.00000
266.00000 546.00000
191.00000 499.00000
398.00000 644.00000
288.00000 615.00000
IMAGE=28751_A_AgkPis_female_2X_scale.tif
ID=28751_A_AgkPis
SCALE=0.004878
LM=10
738.00000 311.00000
632.00000 318.00000
550.00000 331.00000
465.00000 320.00000
382.00000 423.00000
317.00000 388.00000
445.00000 601.00000
373.00000 556.00000
590.00000 729.00000
491.00000 687.00000
IMAGE=28751_B_AgkPis_female_2X_scale.tif
ID=28751_B_AgkPis
SCALE=0.004878
LM=10
468.00000 240.00000
368.00000 255.00000
352.00000 294.00000
284.00000 344.00000
303.00000 436.00000
219.00000 436.00000
378.00000 526.00000
298.00000 486.00000
473.00000 620.00000
403.00000 554.00000
IMAGE=28751_D_AgkPis_female_2X_scale.tif

ID=28751_D_AgkPis
SCALE=0.004877
LM=10
439.00000 211.00000
340.00000 183.00000
251.00000 289.00000
168.00000 281.00000
111.00000 427.00000
51.00000 443.00000
215.00000 591.00000
139.00000 594.00000
369.00000 732.00000
274.00000 713.00000
IMAGE=28754_A_AgkPis_female_2X_scale.tif
ID=28754_A_AgkPis
SCALE=0.004878
LM=10
606.00000 310.00000
506.00000 296.00000
491.00000 420.00000
420.00000 453.00000
465.00000 544.00000
400.00000 559.00000
549.00000 623.00000
480.00000 659.00000
725.00000 653.00000
645.00000 718.00000
IMAGE=28759_A_ColCon_female_2X_scale.tif
ID=28759_A_ColCon
SCALE=0.004878
LM=10
621.00000 305.00000
527.00000 314.00000
518.00000 389.00000
439.00000 411.00000
475.00000 505.00000
387.00000 517.00000
572.00000 576.00000
494.00000 587.00000
723.00000 593.00000
629.00000 636.00000
IMAGE=28759_B_ColCon_female_2X_scale.tif
ID=28759_B_ColCon
SCALE=0.004878
LM=10
610.00000 368.00000
533.00000 324.00000
450.00000 406.00000

379.00000 414.00000
344.00000 504.00000
273.00000 502.00000
414.00000 628.00000
346.00000 601.00000
558.00000 749.00000
480.00000 694.00000
IMAGE=28759_C_ColCon_female_2X_scale.tif
ID=28759_C_ColCon
SCALE=0.004878
LM=10
527.00000 417.00000
425.00000 434.00000
434.00000 540.00000
358.00000 515.00000
367.00000 629.00000
292.00000 635.00000
460.00000 687.00000
392.00000 732.00000
573.00000 760.00000
476.00000 812.00000
IMAGE=28761_A_NerCla_female_2X_scale.tif
ID=28761_A_NerCla
SCALE=0.004878
LM=10
391.00000 413.00000
324.00000 420.00000
275.00000 504.00000
220.00000 526.00000
222.00000 616.00000
174.00000 634.00000
316.00000 683.00000
256.00000 696.00000
455.00000 720.00000
387.00000 752.00000
IMAGE=28761_B_NerCla_female_2X_scale.tif
ID=28761_B_NerCla
SCALE=0.004878
LM=10
530.00000 173.00000
414.00000 174.00000
292.00000 286.00000
204.00000 318.00000
212.00000 490.00000
139.00000 507.00000
294.00000 643.00000
202.00000 645.00000
525.00000 754.00000

412.00000 789.00000
IMAGE=28761_C_NerCla_female_2X_scale.tif
ID=28761_C_NerCla
SCALE=0.004878
LM=10
654.00000 302.00000
546.00000 306.00000
471.00000 382.00000
392.00000 353.00000
344.00000 465.00000
282.00000 454.00000
422.00000 556.00000
340.00000 575.00000
607.00000 676.00000
500.00000 682.00000
IMAGE=28763_A_ColCon_female_2X_scale.tif
ID=28763_A_ColCon
SCALE=0.004878
LM=10
643.00000 400.00000
547.00000 423.00000
525.00000 510.00000
464.00000 546.00000
499.00000 611.00000
446.00000 655.00000
611.00000 678.00000
543.00000 724.00000
789.00000 721.00000
688.00000 750.00000
IMAGE=28763_B_ColCon_female_2X_scale.tif
ID=28763_B_ColCon
SCALE=0.004878
LM=10
507.00000 392.00000
415.00000 366.00000
358.00000 444.00000
286.00000 475.00000
301.00000 536.00000
235.00000 567.00000
383.00000 619.00000
304.00000 640.00000
535.00000 674.00000
440.00000 713.00000
IMAGE=28763_C_ColCon_female_2X_scale.tif
ID=28763_C_ColCon
SCALE=0.004878
LM=10
503.00000 318.00000

395.00000 348.00000
347.00000 450.00000
265.00000 490.00000
255.00000 620.00000
196.00000 633.00000
381.00000 703.00000
293.00000 726.00000
559.00000 756.00000
453.00000 783.00000
IMAGE=29392_A_PytMol_female_2X_scale.tif
ID=29392_A_PytMol
SCALE=0.004878
LM=10
575.00000 394.00000
470.00000 372.00000
416.00000 442.00000
331.00000 441.00000
280.00000 513.00000
227.00000 495.00000
354.00000 669.00000
281.00000 634.00000
498.00000 763.00000
388.00000 775.00000
IMAGE=29392_B_PytMol_female_2X_scale.tif
ID=29392_B_PytMol
SCALE=0.004878
LM=10
609.00000 372.00000
512.00000 361.00000
482.00000 418.00000
397.00000 418.00000
346.00000 475.00000
299.00000 445.00000
414.00000 587.00000
339.00000 555.00000
544.00000 635.00000
445.00000 655.00000
IMAGE=29394_A_PytMol_female_2X_scale.tif
ID=29394_A_PytMol
SCALE=0.004878
LM=10
652.00000 409.00000
556.00000 417.00000
482.00000 488.00000
402.00000 497.00000
382.00000 599.00000
322.00000 597.00000
485.00000 724.00000

405.00000 703.00000
674.00000 812.00000
578.00000 821.00000
IMAGE=29394_B_PytMol_female_2X_scale.tif
ID=29394_B_PytMol
SCALE=0.004878
LM=10
422.00000 289.00000
366.00000 222.00000
268.00000 348.00000
191.00000 330.00000
151.00000 431.00000
86.00000 429.00000
268.00000 528.00000
191.00000 536.00000
419.00000 570.00000
348.00000 642.00000
IMAGE=29402_A_PytMol_female_2X_scale.tif
ID=29402_A_PytMol
SCALE=0.004878
LM=10
719.00000 355.00000
623.00000 307.00000
496.00000 385.00000
423.00000 350.00000
341.00000 430.00000
288.00000 401.00000
411.00000 589.00000
337.00000 554.00000
543.00000 765.00000
445.00000 732.00000
IMAGE=29413_A_PytMol_female_2X_scale.tif
ID=29413_A_PytMol
SCALE=0.004878
LM=10
492.00000 384.00000
403.00000 322.00000
285.00000 399.00000
202.00000 403.00000
139.00000 494.00000
82.00000 478.00000
224.00000 630.00000
140.00000 616.00000
374.00000 785.00000
267.00000 777.00000
IMAGE=29413_B_PytMol_female_2X_scale.tif
ID=29413_B_PytMol
SCALE=0.004878

LM=10
650.00000 212.00000
539.00000 244.00000
501.00000 419.00000
429.00000 463.00000
413.00000 603.00000
349.00000 622.00000
582.00000 724.00000
501.00000 729.00000
822.00000 803.00000
709.00000 819.00000
IMAGE=29413_C_PytMol_female_2X_scale.tif
ID=29413_C_PytMol
SCALE=0.004878
LM=10
707.00000 258.00000
591.00000 226.00000
480.00000 317.00000
387.00000 287.00000
285.00000 399.00000
212.00000 392.00000
352.00000 602.00000
262.00000 606.00000
536.00000 762.00000
420.00000 798.00000
IMAGE=40091_A_ThaSir_female_2X_scale.tif
ID=40091_A_ThaSir
SCALE=0.004878
LM=10
546.00000 328.00000
449.00000 257.00000
348.00000 342.00000
275.00000 287.00000
201.00000 396.00000
138.00000 361.00000
272.00000 571.00000
179.00000 536.00000
392.00000 734.00000
276.00000 703.00000
IMAGE=40091_B_ThaSir_female_2X_scale.tif
ID=40091_B_ThaSir
SCALE=0.004878
LM=10
508.00000 221.00000
396.00000 225.00000
358.00000 347.00000
281.00000 381.00000
272.00000 488.00000

205.00000 502.00000
410.00000 603.00000
322.00000 617.00000
582.00000 685.00000
481.00000 733.00000
IMAGE=40103_A_ThaSir_female_2X_scale.tif
ID=40103_A_ThaSir
SCALE=0.004878
LM=10
533.00000 343.00000
444.00000 273.00000
368.00000 368.00000
284.00000 348.00000
224.00000 453.00000
154.00000 433.00000
292.00000 599.00000
208.00000 576.00000
420.00000 712.00000
312.00000 755.00000
IMAGE=40103_B_ThaSir_female_2X_scale.tif
ID=40103_B_ThaSir
SCALE=0.004878
LM=10
683.00000 340.00000
583.00000 312.00000
510.00000 368.00000
422.00000 365.00000
363.00000 455.00000
294.00000 423.00000
419.00000 607.00000
332.00000 586.00000
555.00000 747.00000
452.00000 712.00000
IMAGE=40104_A_ThaSir_female_2X_scale.tif
ID=40104_A_ThaSir
SCALE=0.004878
LM=10
509.00000 255.00000
409.00000 239.00000
340.00000 361.00000
263.00000 364.00000
240.00000 491.00000
157.00000 481.00000
349.00000 610.00000
262.00000 614.00000
513.00000 656.00000
406.00000 695.00000
IMAGE=40871_A_NerCla_female_2X_scale.tif

ID=40871_A_NerCla
SCALE=0.004878
LM=10
625.00000 296.00000
512.00000 338.00000
419.00000 425.00000
329.00000 448.00000
260.00000 624.00000
190.00000 634.00000
482.00000 685.00000
394.00000 714.00000
721.00000 708.00000
614.00000 722.00000
IMAGE=40871_B_NerCla_female_2X_scale.tif
ID=40871_B_NerCla
SCALE=0.004878
LM=10
546.00000 227.00000
432.00000 229.00000
362.00000 312.00000
277.00000 304.00000
230.00000 427.00000
159.00000 421.00000
343.00000 579.00000
255.00000 570.00000
526.00000 685.00000
414.00000 703.00000
IMAGE=40871_C_NerCla_female_2X_scale.tif
ID=40871_C_NerCla
SCALE=0.004878
LM=10
490.00000 101.00000
374.00000 97.00000
315.00000 201.00000
224.00000 208.00000
217.00000 319.00000
148.00000 329.00000
330.00000 441.00000
249.00000 421.00000
517.00000 506.00000
413.00000 511.00000
IMAGE=40871_D_NerCla_female_2X_scale.tif
ID=40871_D_NerCla
SCALE=0.004878
LM=10
475.00000 385.00000
369.00000 369.00000
293.00000 486.00000

223.00000 469.00000
198.00000 593.00000
135.00000 589.00000
304.00000 699.00000
222.00000 709.00000
492.00000 760.00000
401.00000 808.00000
IMAGE=40871_E_NerCla_female_2X_scale.tif
ID=40871_E_NerCla
SCALE=0.004878
LM=10
591.00000 321.00000
484.00000 294.00000
427.00000 383.00000
346.00000 410.00000
314.00000 537.00000
239.00000 526.00000
420.00000 680.00000
334.00000 655.00000
563.00000 800.00000
460.00000 781.00000
IMAGE=40871_J_NerCla_female_2X_scale.tif
ID=40871_J_NerCla
SCALE=0.004878
LM=10
742.00000 273.00000
631.00000 275.00000
562.00000 375.00000
476.00000 355.00000
434.00000 503.00000
363.00000 503.00000
553.00000 640.00000
459.00000 642.00000
749.00000 732.00000
629.00000 725.00000
IMAGE=40871_K_NerCla_female_2X_scale.tif
ID=40871_K_NerCla
SCALE=0.004878
LM=10
401.00000 300.00000
340.00000 239.00000
249.00000 363.00000
167.00000 345.00000
132.00000 452.00000
65.00000 446.00000
251.00000 545.00000
173.00000 557.00000
399.00000 584.00000

338.00000 664.00000
IMAGE=29402_A_PytMol_female2_2X_scale.tif
ID=29402_A_PytMol
SCALE=0.004878
LM=10
454.00000 268.00000
424.00000 171.00000
291.00000 314.00000
221.00000 286.00000
185.00000 395.00000
125.00000 382.00000
273.00000 478.00000
223.00000 513.00000
414.00000 559.00000
359.00000 636.00000
IMAGE=29402_B_PytMol_female_2X_scale.tif
ID=29402_B_PytMol
SCALE=0.004878
