# Supplementary material for: Host-specific phenotypic variation of a parasite co-introduced with invasive Burmese pythons
Source: PLoS One. 2019 Jan 2;14(1):e0209252. doi: 10.1371/journal.pone.0209252 (PMC6314578; doi:10.1371/journal.pone.0209252)
Supplement: S2 File — The catalog number is provided for Raillietiella orientalis pentastomes examined using geometric morphometric analyses. All R. orientalis specimens were deposited to the Auburn University Museum of Natural History (AUM). The host species of each parasite is provided. Pentastomes from the same host share the same catalog number with different individual parasites identified by letter. (PDF) [file pone.0209252.s002.pdf]

Supplemental Data S2. The catalog number is provided for *Raillietiella orientalis* pentastomes examined using geometric morphometric analyses. All *R. orientalis* specimens were deposited to the Auburn University Museum of Natural History (AUM). The host species of each parasite is provided. Pentastomes from the same host share the same catalog number with different individual parasites identified by letter.

AUM Catalog Number	Host Species
28744 A	*Nerodia clarkii*
28744 B	*Nerodia clarkii*
28744 C	*Nerodia clarkii*
28744 D	*Nerodia clarkii*
28761 A	*Nerodia clarkii*
28761 B	*Nerodia clarkii*
28761 C	*Nerodia clarkii*
40871 A	*Nerodia clarkii*
40871 B	*Nerodia clarkii*
40871 C	*Nerodia clarkii*
40871 D	*Nerodia clarkii*
40871 E	*Nerodia clarkii*
40871 J	*Nerodia clarkii*
40871 K	*Nerodia clarkii*
28751 A	*Agkistrodon piscivorus*
28751 B	*Agkistrodon piscivorus*
28751 D	*Agkistrodon piscivorus*
28754 A	*Agkistrodon piscivorus*
28759 A	*Coluber constrictor*
28759 B	*Coluber constrictor*
28759 C	*Coluber constrictor*
28763 A	*Coluber constrictor*
28763 B	*Coluber constrictor*
28763 C	*Coluber constrictor*
40091 A	*Thamnophis sirtalis*
40091 B	*Thamnophis sirtalis*
40103 A	*Thamnophis sirtalis*
40103 B	*Thamnophis sirtalis*
40104 A	*Thamnophis sirtalis*
29392 A	*Python bivittatus*
29392 B	*Python bivittatus*
29394 A	*Python bivittatus*
29394 B	*Python bivittatus*
29402 A	*Python bivittatus*
29413 A	*Python bivittatus*
29413 B	*Python bivittatus*
29413 C	*Python bivittatus*
29402 A	*Python bivittatus*
29402 B	*Python bivittatus*
